# Behavioral mapping of multisensory experience and engagement in accessible gardens: a case study of Xuanwu Lake Park, Nanjing

**DOI:** 10.3389/fpsyg.2026.1733848

**Published:** 2026-04-09

**Authors:** Ying Sun, Xiangfeng Li, Zhizhe Sun, Mo Zhou, Zhixin Xu

**Affiliations:** 1 School of Architecture, Southeast University, Nanjing, China; 2 School of Architecture and Planning, Hunan University, Changsha, China; 3 College of Landscape Architecture, Zhejiang A&F University, Hangzhou, China

**Keywords:** accessible gardens, behavioral maps, health equity, multi-sensory experiences, perceptual behaviors, visually impaired people

## Abstract

Numerous studies have shown that sensory stimulation in urban gardens can help improve people's behavioral performance and physical and mental health. Accessible gardens for people with visual impairments provide crucial settings for sensing outdoor environments and may support social integration and psychological restoration. This study uses the Xuanwu Lake Accessible Garden in Nanjing as a case. Users' perceptual behaviors were systematically documented through behavioral observation. Demographic and behavioral data for 1,167 users were recorded on base maps and grid sheets. Daily patterns of use were further stratified by behavior categories and users' profiles, and their relationship with the sensory-space type and behaviors was assessed with Chi-square test. In parallel, 15 semi-structured interviews with visually impaired participants were conducted under the Sensory Experience Enlightenment Protocol (SEEP), and thematic analysis in NVivo was used to characterize their positive and negative sensory experiences in the garden. Results indicated that the multisensory spaces in the accessible garden provided rich, discernible stimuli, and sensory elements were significantly associated with users' perceptual behaviors. Overall experiences among visually impaired participants were positive, with tactile and auditory dimensions most salient. This study provides empirical evidence to inform multisensory design in accessible gardens by identifying how sensory-space features are associated with behavioral engagement and self-reported experiences, thereby suggesting design opportunities that may support restorative experiences and social participation.

## 1 Introduction

As an integral component of the urban green space system, accessible sensory gardens are increasingly recognized as critical venues for promoting human physical and mental health while facilitating interaction with nature (Ekkel and de Vries, 2017). In recent years, a growing number of public spaces, community green areas, and healthcare facilities have incorporated garden spaces into their design to enhance residents' wellbeing and overall quality of life (Wang et al., 2019). Due to their focus on the diverse needs of users, accessible sensory gardens have been described using various terms, including sensory gardens, therapeutic gardens, and restorative gardens (Szabo et al., 2023). Beyond emphasizing the restorative potential of nature, they also embody principles of environmental inclusiveness and social functionality.

From the perspective of sensory experience, accessible sensory gardens employ Universal Design principles and multi-sensory stimulation to create rich, interactive environments (Hussein et al., 2016). These spaces support not only visual, auditory, olfactory, and tactile experiences but also emphasize users' behavioral interactions with both the garden and the natural elements within it. Previous studies indicate that environmental perception directly influences public spatial behavior and attitudes, and that the quality of sensory stimuli together with environmental structure significantly enhances satisfaction and encourages restorative activities. Therefore, understanding the relationship between environmental characteristics and users' perceptual behaviors (i.e., observable behavior patterns through which users attend to or engage with sensory cues in the garden), and identifying design elements that can enhance multi-sensory experiences, has become an important direction for optimizing the development of urban accessible sensory gardens.

Meanwhile, increasing attention has been paid to restorative environments, which emphasize the positive role of natural spaces in stress regulation and psychological recovery (Ulrich, 2002). As one of the most accessible multi-sensory public spaces for residents, urban accessible sensory gardens hold significant restorative potential. This is particularly relevant for people with disabilities, as their multi-sensory interventions align more closely with the specific requirements of environmental experience and contribute to improvements in both physical and mental health (Ojala et al., 2019; Suci and Sukarno, 2021). In recent years, the concept of accessible gardens has gradually expanded from an initial focus on auditory-friendly design (Krzeptowska-Moszkowicz et al., 2021; Pawłowska, 2008) to broader universal design strategies involving path layout and interactive facilities (Zajadacz and Lubarska, 2020), while progressively incorporating multi-sensory dimensions such as touch, smell, and soundscape (Diehl, 2024; Chen, 2008; Pan et al., 2015). Emerging research has also begun to address perceptual differences among specific groups, such as people with visual impairments, and to propose corresponding design strategies and theoretical frameworks. Despite these advances, current studies have only preliminarily revealed the potential value of multi-sensory gardens, and systematic research on the perceptual behaviors of actual users remains limited. In particular, there is still insufficient empirical evidence regarding how individuals interact with sensory spaces in real-life contexts, how spatial elements influence behavioral responses, and how perceptual experiences feed back into design strategies. Therefore, it is urgent to adopt a behavioral perspective to explore the dynamic relationship between users and accessible garden spaces, thereby enriching the empirical foundation for sensory space design.

In this context, the present study focuses on the multi-sensory experience of accessible gardens and aims to explore the relationship between such spaces and users' perceptual behaviors. Specifically, it seeks to address the following questions:

■ What are the characteristics of the user groups in accessible gardens, including “how many people,” “where they are,” and “how they use the space?”
■ How are the sensory space attributes of accessible gardens associated with users' perceptual behaviors?
■ For whom are accessible gardens designed, and what practical performance and unmet needs are observed in real use (with a focus on visually impaired users)?
■ How can sensory stimuli be better utilized to provide positive perceptual experiences for all users?

We first characterize user composition, spatial distribution, and use patterns in the accessible garden through behavioral mapping (*N* = 1,167), establishing a descriptive baseline for subsequent analyses. Building on this baseline, this study seeks to advance understanding of users' experiences and needs in accessible sensory gardens, to derive evidence-informed recommendations for the sustainable design of multisensory urban accessible gardens, and to promote more equitable everyday recreational participation for people with visual impairments.

## 2 Methods

### 2.1 Study area

**Figure 1:**
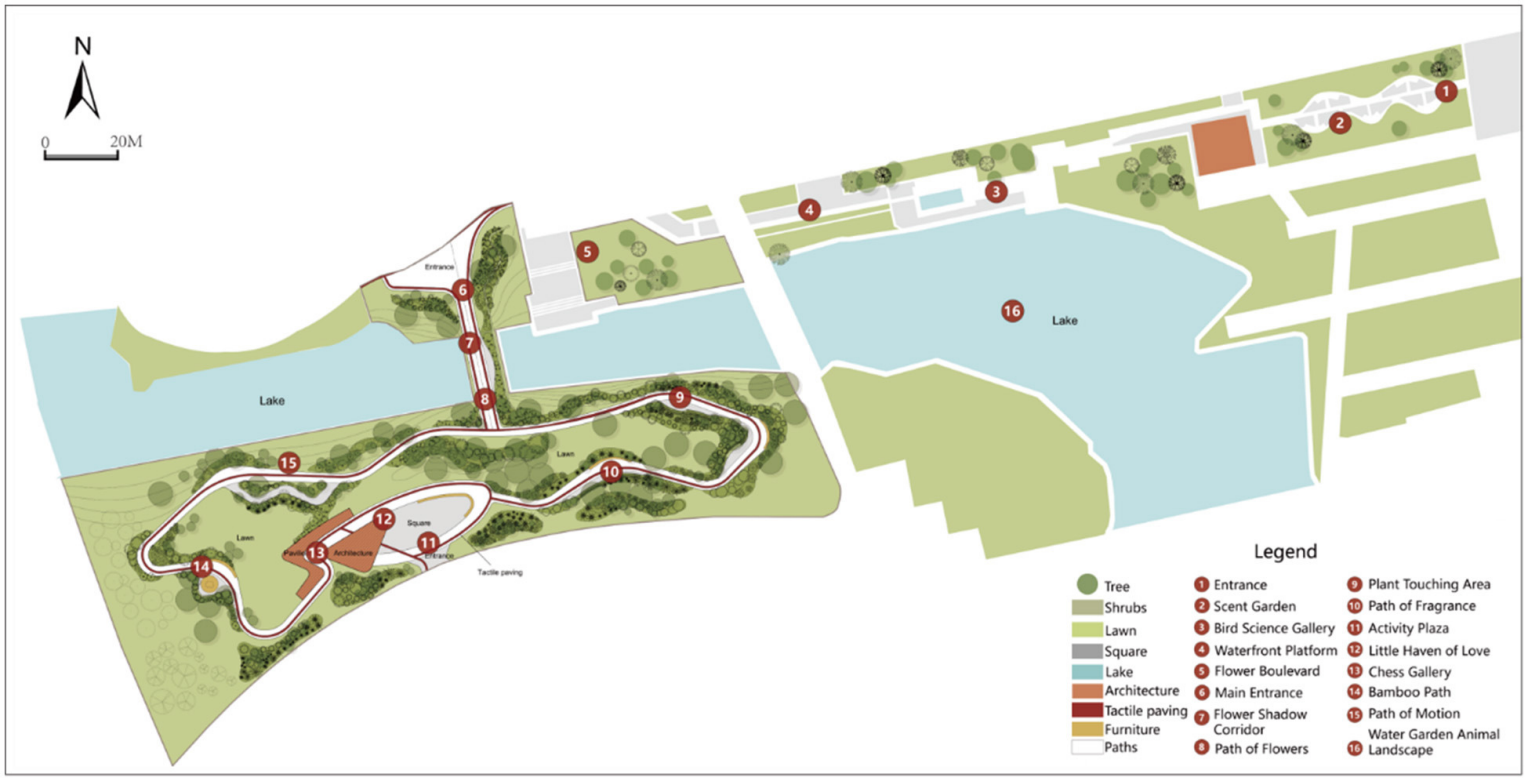
Accessible garden plan.

The study focuses on the accessible sensory garden at Xuanwu Lake in Nanjing, which is one of the first demonstration projects in Nanjing's 2024 “Accessible Construction Pilot Zone.” The garden, covering an area of approximately 8,000 square meters, has been designed with full consideration for the recreational needs of people with disabilities, the elderly, and other special groups. It consists of two main areas: the peripheral zone and the core zone. The peripheral zone extends the core area's accessible walkway system to the main entrance of the scenic area, seamlessly integrating with the city's accessible infrastructure. The core zone primarily serves individuals with visual impairments, featuring a 420-meter circular path and various accessible facilities to guide visitors with visual disabilities. Additionally, the garden is equipped with sensory experience facilities, such as a birdwatching corridor, plant touch displays, and tree trunk chimes, encouraging the use of auditory, olfactory, and tactile experiences for visually impaired visitors (Figure 1).

**Figure 2:**
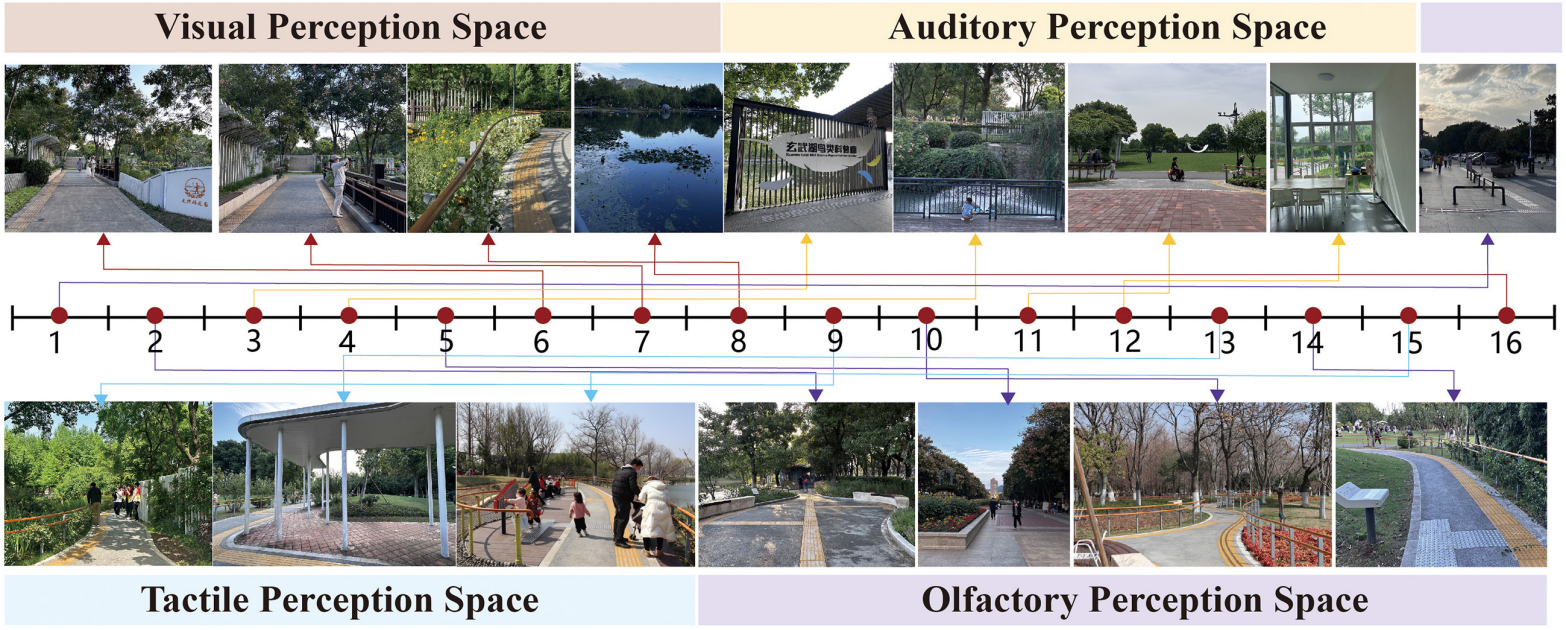
Accessible garden sensory space spot map.

This study employs a literature review, field analysis, observation of garden user groups, and basic interviews with individuals with visual impairments to explore the perceptual space of accessible sensory gardens. Focusing on the expressive layer of landscape elements (those perceptible and measurable by users), such as the fundamental components of the garden (terrain, architecture, water features, plants, etc.) and natural conditions (wind, sunlight, temperature, humidity, etc.), along with the perceptual layer of individuals with disabilities, the study identifies the perceptual spaces within accessible gardens. These include visual, auditory, olfactory, and tactile sensory spaces (Figure 2). The entrance to the core area of the garden features natural elements such as flowers, water features, and trees, with the overall spatial design guiding users' visual perception. The auditory sensory space is primarily located in the extended area of the garden, pre-dominantly featuring artificial elements such as automatic voice broadcasts of bird calls, voice prompts, wind chimes, and plant audio explanations. The tactile sensory space is primarily concentrated in the core area of the garden, featuring touch zones with plants, organ strikes, drum taps, trampolines, and other tactile experiences. The olfactory sensory space is primarily located in both the extended area and at the exit of the core zone, where 43 distinct aromatic plants, including gardenia, fennel, stevia rebaudiana, and mint, stimulate users' olfactory experiences (Table 1).

**Table 1:** Sensory space classification for accessible gardens.

Spatial type	Perceptual element	Landscape-perception-experience content	Note
Visual perception space	Sight	The central lake view serves as the focal point of the entire garden, offering expansive vistas.	Figure 2. points 6, 7, and 16
	Color block	Plant ornamental plants such as canna lilies, lotus flowers, and cherry blossoms for their rich colors	Figure 2. point 8
	Light	Ground lights provide illumination throughout the garden	Figure 2. points 6, 7, 12
Auditory perception space	Natural elements	Bird songs, insect chirps, dynamic water features (fountains, streams), and the sound of the wind	Figure 2. points 3, 4
	Human-made features	The tinkling of wind chimes, the narration of plant audio guides, the chatter and laughter of the crowd, the sound of footsteps	Figure 2. points 11, 12
Tactile perception space	Using the hand as a medium	Touchable plants (plant touch zone, experience identifying different plants), accessible water features (water-friendly steps), diverse materials (various sculptural ornaments, chess tables and chairs)	Figure 2. points 9, 13
	Using the foot as a medium	Different paving materials (large paved areas, gravel paths), trampolines	Figure 2. point 15
	Using skin as a medium	Changes in temperature, light, and humidity (alterations in temperature and humidity when entering different spaces)	Throughout the entire garden
Olfactory perception space	Plant scents	Floral scents (from growing aromatic plants like peppermint), the scent of foliage, and the aroma of freshly trimmed plants	Figure 2. points 2, 5, 10, 14
	Other odors	The scent of soil, water, animals, food, etc	Figure 2. point 1

### 2.2 Study methods

The study began with an on-site reconnaissance of the accessible garden. Based on the spatial structure and the distribution of facilities, the garden was delineated into a set of functional nodes and observation zones, which served as the basic spatial units for behavioral recording and positioning. Following Fernandes (2017), who recommended covering different seasonal conditions, behavioral observations were conducted on two separate days in 2024 (one in May and one in October), from 09:00 to 18:00, to capture garden use patterns under different seasonal contexts. Field records included users' demographic and travel characteristics (e.g., gender, age group, travel mode, and use of mobility aids), perceptual behavior type, and the node in which the behavior occurred, together with the corresponding time period and typical activity locations. To enhance consistency and objectivity, all observers received standardized training, and data collection was organized by zoning responsibilities to avoid overlap; records were subsequently cross-checked.

For behavioral observation and behavioral mapping, we followed the behavior-mapping procedure proposed by Ittelson et al. (1970). A perceptual behavior classification and coding scheme was established prior to systematic observation. Each observed event was assigned to a pre-defined node as the positional unit in the field. After fieldwork, a node layer was created in ArcGIS Pro based on the garden base plan, and observation records were digitized and visualized according to these spatial units. The positional accuracy of the behavioral maps should therefore be interpreted as node-level positional accuracy: events were geocoded to pre-defined functional node/zone (rather than an exact GPS coordinate), meaning that mapped locations are accurate at the level of the node boundary (or a representative point/centroid). Thus, the maps reflect node-level spatial patterns rather than sub-meter positional measurements. Symbolization rules were pre-defined in ArcGIS to ensure consistency and replicability: symbol shape denotes age group (children/adolescents, middle-aged adults, and older adults), symbol fill (hollow vs. solid) distinguishes independent visits from group visits, and symbol color indicates perceptual behavior type (passage, leisure/staying, passive perception, active perception, and social interaction).

**Figure 3:**
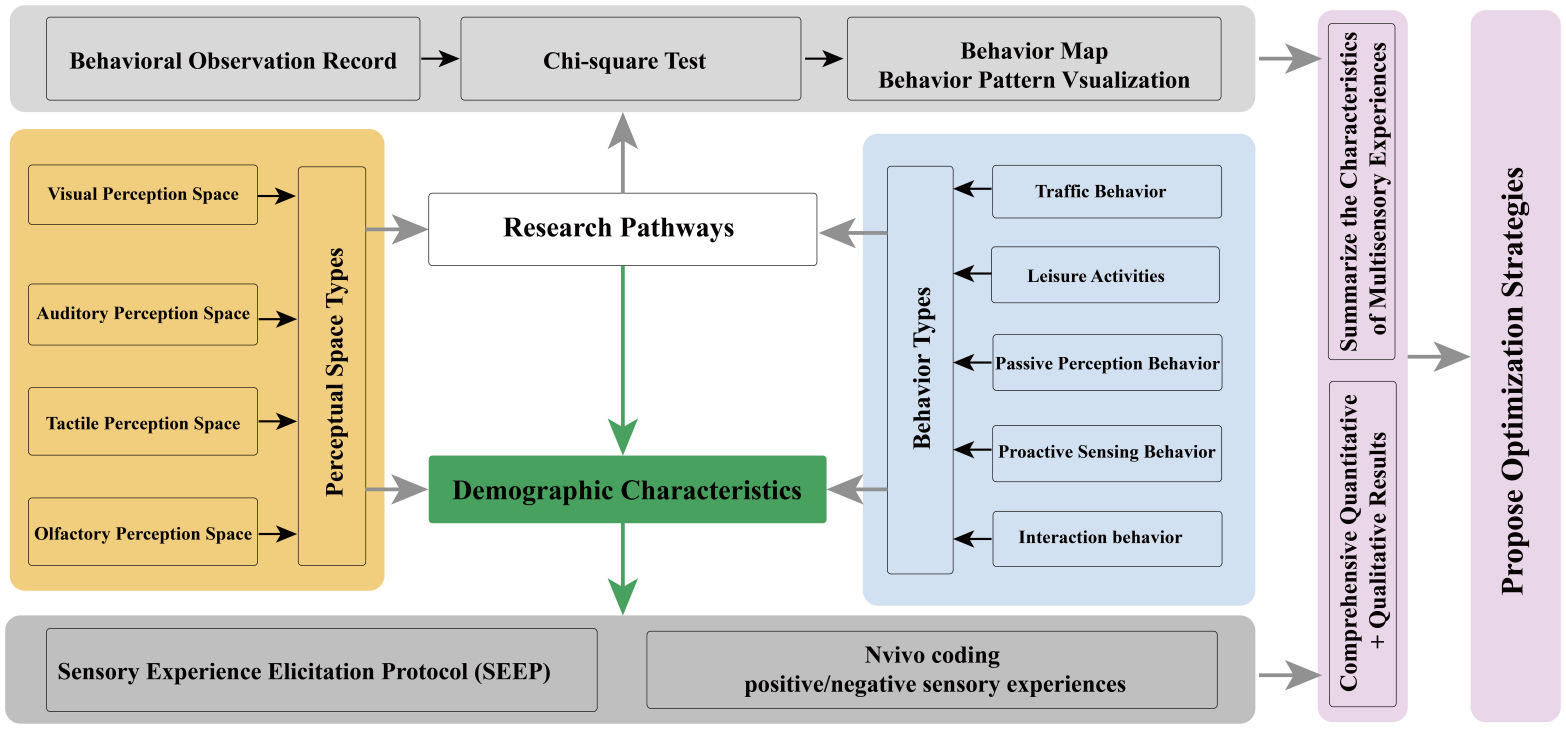
Research methodology and logical framework.

In addition, Chi-square tests were conducted in SPSS 25.0 to examine associations between perceptual-space types and users' demographic/travel characteristics and perceptual behaviors. Given that visually impaired individuals constitute a key target group of accessible gardens, we further recruited visually impaired participants (*N* = 15) through the Nanjing Association for the Blind. Semi-structured interviews were conducted using the Sensory Exchange Experience Protocol (SEEP) (Gretzel and Fesenmaier, 2010), and qualitative coding and thematic synthesis were performed in NVivo 12 (Corbin and Strauss, 2014) to extract positive and negative sensory experience elements across perceptual spaces (Table 2). Finally, ArcGIS Pro was used to spatially represent the locational references of these experience elements. Building on the above analyses of general users' perceptual behaviors and visually impaired participants' multisensory experiences, we propose targeted strategies to enhance positive perceptual experiences in accessible gardens (Figure 3).

**Table 2:** SEEP issues.

Question	Sensory stimulation (sensory)/code
Before arriving at the garden, please describe your impressions and feelings about the accessible garden.	The most important impression (overall perception and feedback) Local associations (SEEP 6–7)
What color makes the deepest impression on you? (For people with low vision)	Psychological implications of color design (Visual; SEEP1)
After touring the accessible garden, what scene or element left the deepest impression on you?	Acoustics (auditory; SEEP2)
What type of voice leaves the deepest impression on you?	Acoustics (auditory; SEEP2)
What is the scent that leaves the deepest impression on you?	Olfactory (smell; SEEP3)
What is the texture that leaves the deepest impression on you?	Tactile (touch; SEEP5)
After the tour, what images come to mind when you think of this place?	(After the visit) primary perceptions (overall perceptions and feedback; SEEP 6–7)

## 3 Results

### 3.1 User behavior types

**Figure 4:**
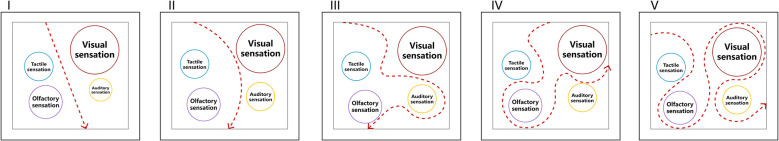
Schematic diagram of types of perceptual behavior.

In the initial stage of the study, a preliminary investigation of the accessible sensory garden was conducted to observe visitor behaviors and to describe categories of users' perceptual behaviors within the garden. Based on the results of this pre-investigation, user perceptual experiences were classified into five categories: transit behavior (Moudon, 1994), leisure behavior, passive perceptual behavior, active perceptual behavior (Cepić et al., 2024), and social interaction behavior (Figure 4). Four observational indicators were applied to differentiate these behavior types: (1) users remained in the garden space for an extended period of time; (2) users showed clear interest in the garden facilities; (3) users' facial expressions indicated a state of positive engagement; and (4) users touched sensory installations in the garden, triggering sound or other perceptual responses.

First, transit behavior refers to users passing through the garden along the shortest path while remaining in a state of unconscious perception of the space. Leisure behavior refers to users who primarily engage in visual sensory experiences. Passive perceptual behavior refers to users in a passive perceptual state, perceiving the garden merely as an urban green space suitable for walking and unconsciously experiencing sensory stimuli such as scents and sounds. Active perceptual behavior refers to users who deliberately choose to stay in the garden for a relatively long period of time and show interest in specific sensory experiences. Social interactive behavior refers to users engaging in multi-sensory experiences, becoming fully immersed in the perceptual space of the accessible garden. Detailed descriptions of these categories are provided in Table 3.

**Table 3:** Five levels of perceptual behavior classification.

Behavior type	Behavioral characteristics description	Sensory modalities involved (ranked by stimulus intensity)
I) Transit behavior (Vidal et al., 2022)	Take the shortest path through the garden without making eye contact	None
II) Leisure behavior (Wang et al., 2024)	Slow walking, sitting, or lingering within the garden without direct engagement with specific sensory installations or elements (i.e., no observable sensory-focused actions such as smelling, touching, listening/attending to a sound source). Users may pause briefly for rest or conversation, but do not show explicit exploratory interaction with sensory features.	Sight
III) Passive perceptual behavior (Li et al., 2019)	Users remain stationary or move slowly while exhibiting observable sensory reception (e.g., pausing to look toward a feature, listening to water/sound sources, or inhaling near fragrant vegetation) without direct manipulation of sensory elements (e.g., no touching/operating installations). This category captures passive noticing of sensory cues rather than active exploration.	Smell/sight/hearing
IV) Active perceptual behavior (Han et al., 2022)	Pause briefly in the garden space, listen to the sound of the fountain, look around, and inhale the fragrance.	Touch, smell, sight, hearing—individually or in cross-modal combinations
V) Social interaction behavior (Wang et al., 2021; Ngesan et al., 2013)	Children interact with sensory-based installations—touching plants, running through installations, and striking wind chimes.	Two or more sensory experiences
	Users come to the garden daily for rehabilitation exercises.	
	In the garden, reclining on a wooden chair amidst the bamboo grove.	
	Perceiving every space within the garden	

To minimize observer subjectivity during non-participant observation, Level II and Level III were distinguished using observable proxies.

Level II was coded when users showed leisure-oriented movement or lingering without identifiable sensory-focused actions.

Level III was coded when users displayed sensory-attending cues (e.g., pausing and orienting the head/body toward a sensory feature, brief scanning, or inhaling near fragrant vegetation) but without direct manipulation of sensory elements.

Dwell time (short stop vs. sustained stay), movement speed (slow vs. transit), and the presence/absence of sensory-oriented actions were used as practical coding cues in the field.

### 3.2 Using crowd sensing for spatial behavior analysis

#### 3.2.1 Perceived spatial user demographic characteristics

A total of 1,167 user data entries were collected through on-site observation of the accessible sensory garden. According to the statistical results (Table 4), the majority of garden users were adult women and older adults (*p* = 0.001). More than half of the users visited the garden in groups (76.35%), while a smaller proportion visited alone (23.65%). Most users had no mobility restrictions (94.77%), with only a small number using assistive devices such as wheelchairs, strollers, or white canes (5.23%). Children primarily engaged with the visual (53.5%) and tactile (15.9%) perceptual spaces, whereas adults and older adults were more likely to enter the visual and auditory spaces.

**Table 4:** Information sheet on demographic characteristics of users of accessible gardens.

Variable	Category	Accessible garden (*n* = 1,167)				*x*^2^; *P*
		**Visual perception space (*****n*** = **1,113)**	**Auditory perception space (*****n*** = **455)**	**Tactile perception space (*****n*** = **295)**	**Olfactory perception space (*****n*** = **386)**	
		***n*** **(%)**	***n*** **(%)**	***n*** **(%)**	***n*** **(%)**	
Gender	Male	547 (53.3)	198 (19.3)	129 (12.6)	152 (14.8)	12.878; 0.004908
	Female	566 (46.3)	257 (21)	166 (13.6)	234 (19.1)	
Age	Children/adolescents	138 (53.5)	39 (15.1)	41 (15.9)	40 (15.5)	60.8588; 0.001
	Adults	709 (54.9)	249 (19.3)	138 (10.7)	195 (15.1)	
	Elderly	266 (38)	167 (23.9)	116 (16.6)	151 (21.6)	
Status	Traveling alone	329 (57.8)	111 (19.5)	54 (9.5)	75 (13.2)	25.5578; < 0.001
	Travel together	784 (46.7)	344 (20.5)	241 (14.3)	311 (18.5)	
Mobility impairment	Restrictions apply (strollers, canes, wheelchairs)	46 (39.7)	29 (25)	16 (13.8)	25 (21.6)	5.20912; 0.1571
	Unrestricted	1,067 (50)	426 (20)	279 (13.1)	360 (16.9)	
Time	Morning (9:00 a.m.−13:00 pm)	507 (47.5)	191 (17.9)	153 (14.3)	216 (20.2)	20.5835; < 0.001
	Afternoon (13:00–19:00)	606 (51.3)	264 (22.3)	142 (12)	170 (14.4)	
Levels of interactive perception in garden engagement	Transit behavior	80 (30.1)	58 (21.8)	76 (28.6)	52 (19.5)	407.076; 0.001
	Leisure behavior	589 (68.8)	98 (11.4)	69 (8.1)	100 (11.7)	
	Passive perceptual behavior	51 (22.3)	64 (27.9)	48 (21)	66 (28.8)	
	Active perceptual behavior	354 (49.6)	136 (19)	87 (12.2)	137 (19.2)	
	Social interactive behavior	39 (21.2)	99 (53.8)	15 (8.2)	31 (16.8)	

A strong statistical association was observed between users and their perceptual behaviors when interacting with the garden (*p* = 0.001). Among them, the visual perceptual space had the largest number of users, but most engaged only in leisure behavior (68.8%) with limited interaction with the accessible garden. The auditory perceptual space generated the highest proportion of social interactive behavior (53.8%), while the olfactory space was mainly associated with passive perceptual behavior (28.8%), and the tactile space was dominated by transit behavior (28.6%).

#### 3.2.2 Perceiving spatial user behavior characteristics

**Figure 5:**
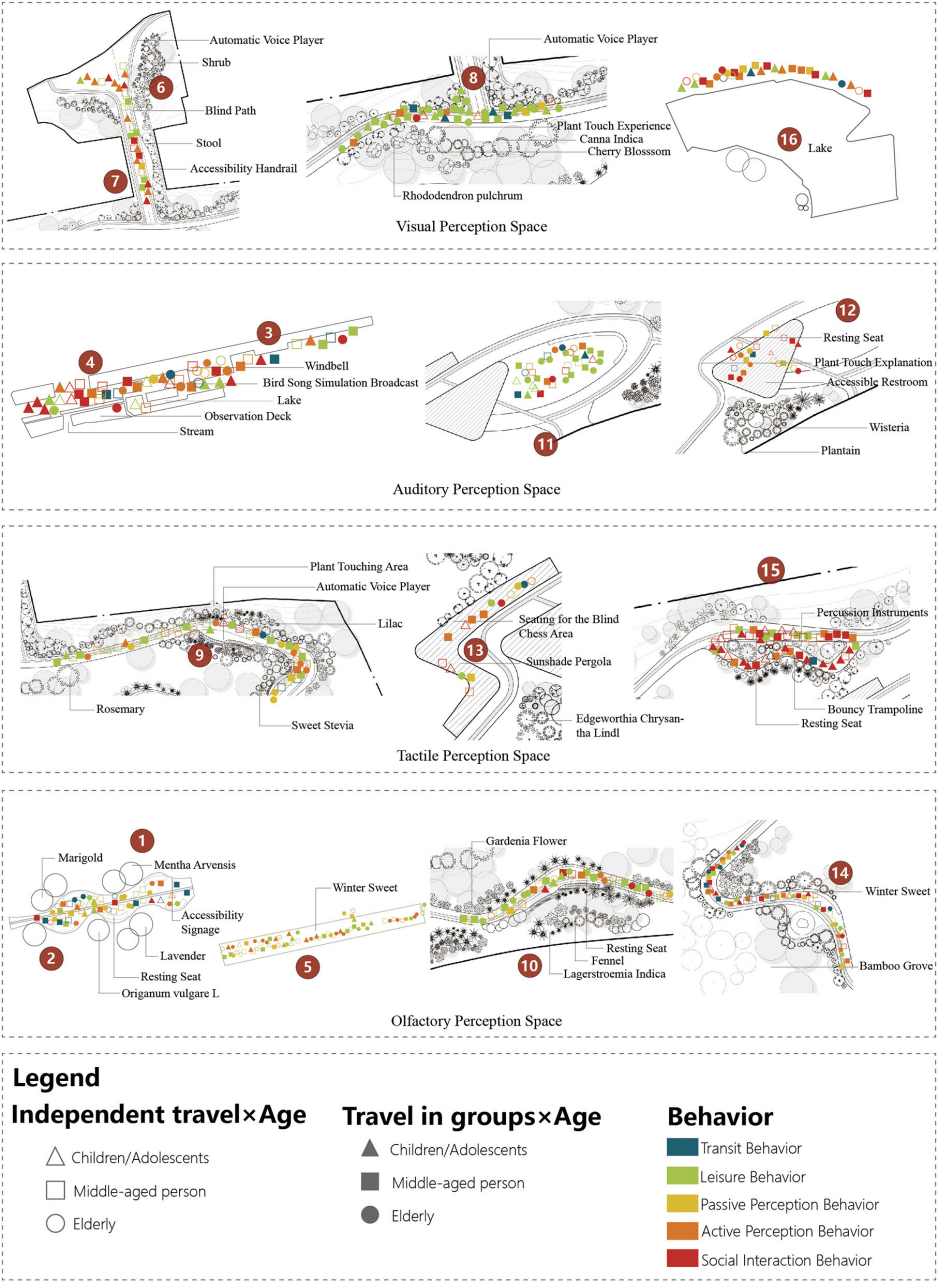
Accessible garden visitor behavior map.

By combining the behavioral maps with the statistical data from Table 4, this study examines how different perceptual spaces within the accessible sensory garden influence user behavior (Figure 5). The primary sensory stimuli in the visual perceptual space include water features, flowers, plants, and animals. This space is pre-dominantly used by adult and older women. Transit and leisure behaviors occur most frequently in the visual perceptual space. The main reasons are as follows: first, in terms of pathways, the visual perceptual space largely overlaps with the circulation routes, enabling users to pass quickly through the accessible garden, which results in the highest number of users in this space. Second, benches and seating areas are provided throughout the visual space, encouraging users to rest, chat, and prolong their stay in the garden.

The auditory perceptual space is primarily characterized by human-made features, including wind chimes and automatic voice broadcasts, supplemented by natural sounds such as fountains and streams. Among the four auditory spaces, Social interactive behaviors were observed most frequently, with older women comprising the largest user group. Because the extended areas of the garden are equipped with sound devices simulating bird calls and other auditory prompts, elderly users tend to engage in perceptual experiences in these spaces.

The tactile perceptual space is used pre-dominantly by children, with the main activity areas located near the trampoline zone and soundscape installations. First, children are naturally attracted to activities involving risk, speed, excitement, stimulation, and uncertainty. To some extent, uneven ground surfaces, lower physical barriers, and landscape elements with tactile qualities stimulate children's sense of adventure and challenge, thereby enhancing their experiential satisfaction (Stephenson, 2003). Second, the American Horticultural Therapy Association (AHTA) defines a therapeutic garden as “a plant-dominated environment designed to facilitate interaction with the healing elements of nature” (Wintherbottom and Wagenfeld, 2015). Stigsdotter and Grahn further suggest that sensory stimulation can strengthen user–garden interactions and help create a therapeutic environment (Stigsdotter and Grahn, 2002; Ulrich et al., 1991). The Xuanwu Lake Accessible Garden includes 43 species of distinctive aromatic plants, which provide restorative qualities and attract certain users seeking sensory and therapeutic experiences.

Studies suggest that some individuals with visual impairments may develop heightened olfactory sensitivity when vision is absent or limited, and that smell can support spatial perception, environmental orientation, memory, and emotional regulation. Consistent with this, during the field investigation, visually impaired participants reported salient olfactory cues from natural elements such as airflow, plants, and trees, while describing unpleasant experiences associated with artificial odors. From the behavioral mapping data, the olfactory perceptual space was pre-dominantly used by adults and older adults, and was characterized mainly by passive perception and short stays. Such behaviors were most observed near the olfactory node at the entrance to the garden's extended area and around the trampoline area in the core zone. Together, these findings indicate that olfactory cues can function as meaningful environmental information for visually impaired users, but inappropriate odor sources may also undermine sensory comfort.

In summary, behavioral mapping indicates that the accessible garden is used primarily by adult women and older adults, with visits typically occurring in groups and largely without mobility constraints. Use patterns show clear spatial differentiation across perceptual-space types, with overall activity concentrated in visual and auditory spaces. Moreover, perceptual interaction patterns vary significantly by perceptual-space type: visual spaces are dominated by passing-through and leisure behaviors, whereas auditory spaces are more frequently associated with social interaction. In contrast, olfactory spaces are characterized mainly by passive perception, while tactile spaces are used disproportionately by children, particularly around the trampoline area and soundscape installations.

### 3.3 Perceiving spatial user behavior characteristics

Fifteen visually impaired participants were recruited through the Nanjing Association for the Blind. Participant characteristics are summarized in Table 5, including gender, age group, visual status (blindness vs. low vision), onset of visual impairment (congenital vs. acquired), and frequency of visits to the accessible garden. Visual status and related characteristics were self-reported. Semi-structured interviews were conducted following the Sensory Exchange Experience Protocol (SEEP) to capture positive and negative sensory experiences and related environmental factors.

**Table 5:** Characteristics of visually impaired interview participants (*N* = 15).

Variable	Category	*n* (%)
Gender	Male	7 (45.67)
	Female	8 (53.33)
Age	40–49	2 (13.33)
	50–59	7 (46.67)
	60–69	4 (26.67)
	70–79	2 (13.33)
Vision	Blindness	6 (40)
	Low vision	9 (60)
Onset of visual impairment	Congenital blindness	9 (60)
	Acquired blindness	6 (40)
Frequency of visits to the accessible garden	Never	3 (20)
	Once a month	4 (26.67)
	Once or twice a week	5 (33.33)
	More than three times a week	3 (20)

#### 3.3.1 Positive and negative perceptual experiences of the visually impaired

**Figure 6:**
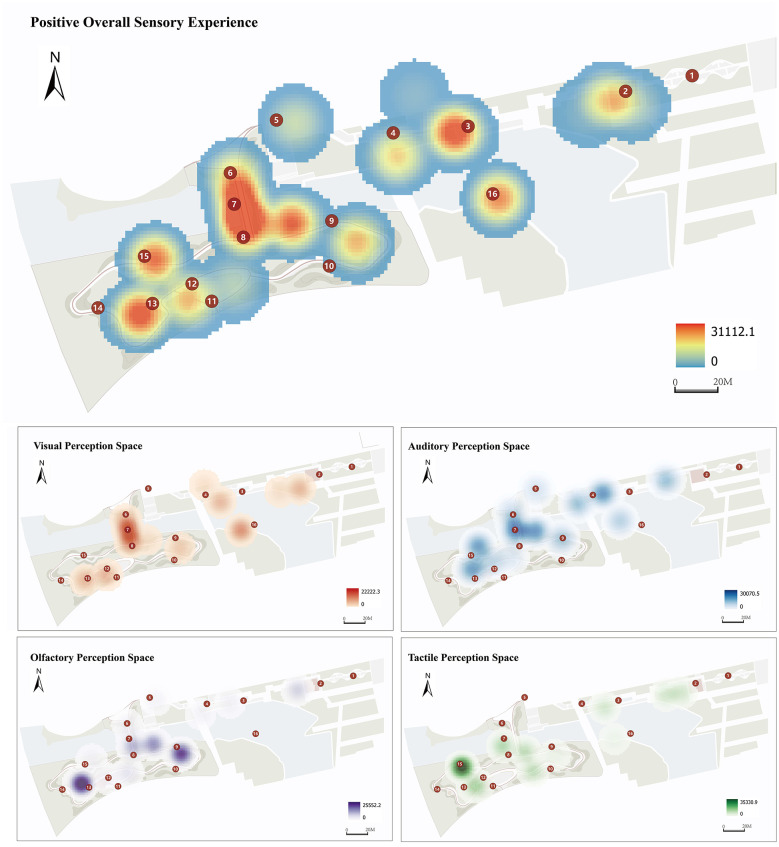
Accessible garden positive sensory experience.

**Figure 7:**
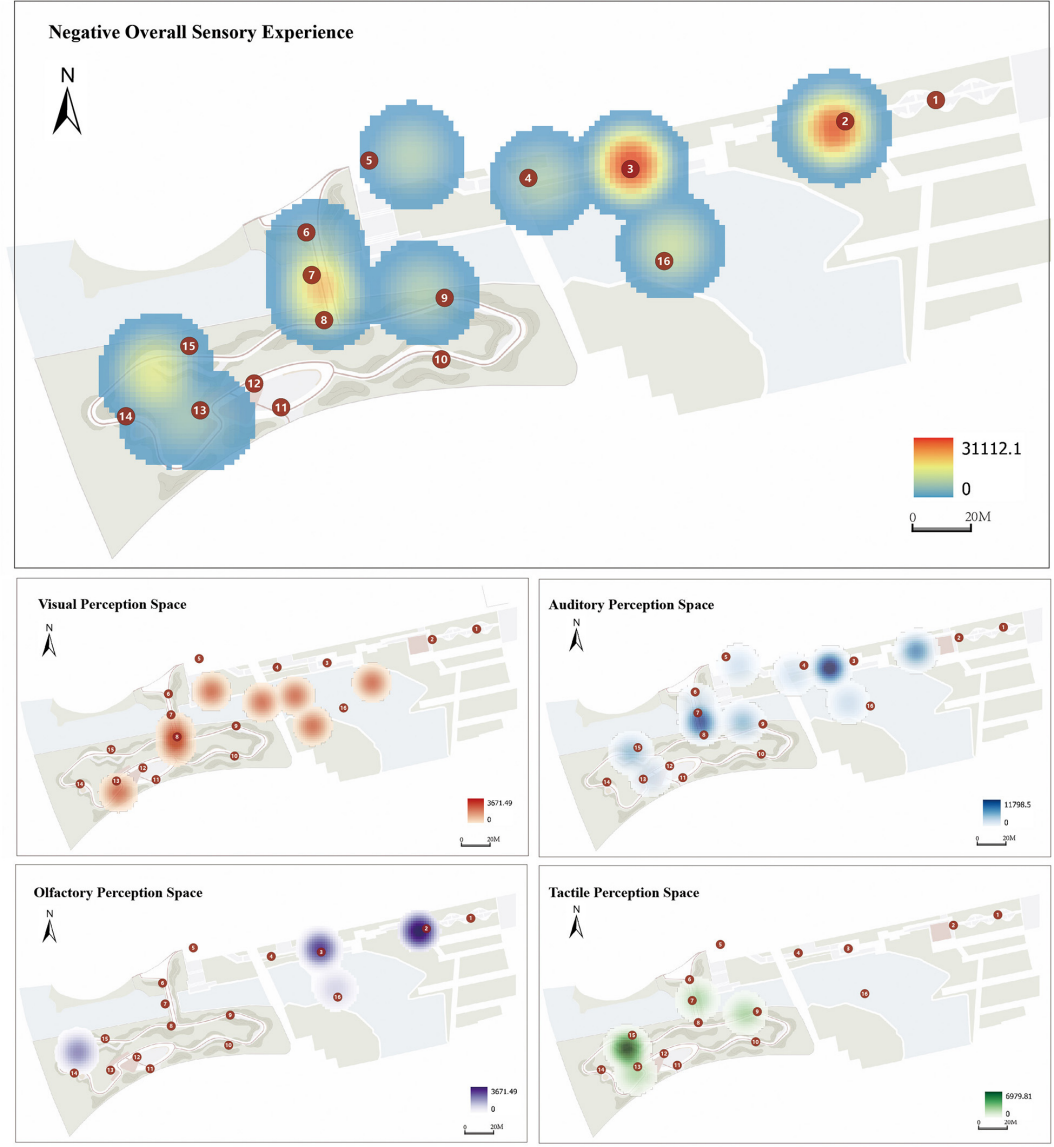
Accessible garden negative sensory experience.

The sensory experiences reported by visually impaired participants were coded into positive and negative categories using NVivo software, with the results presented in Figures 6, 7. Positive experiences were most concentrated around the Bird Science Corridor, with the strongest sensory stimulation recorded at the entrance of the garden's core area. In contrast, the most significant negative sensory experiences were observed at the entrance to the extended area, where the proximity to the East Gate of Xuanwu Lake Park exposed participants to excessive noise, which interfered with their perceptual experience.

From the perspective of the four sensory modalities, most sensory stimuli were concentrated in the core area of the garden and at the entrance to the extended area. As users entered from the extended area, they gradually reported positive olfactory experiences. Approaching the Bird Science Corridor and the entrance to the core zone, participants generally experienced positive auditory stimulation through voice broadcasts. The trampoline activity area in the core zone elicited positive tactile experiences, while positive olfactory experiences were primarily concentrated in the “Bamboo Path” and “Fragrance Path,” where bamboo and aromatic plants were cultivated to encourage visually impaired users to “appreciate” plants through their sense of smell.

#### 3.3.2 Positive and negative experiences of sensory elements among the visually impaired

**Figure 8:**
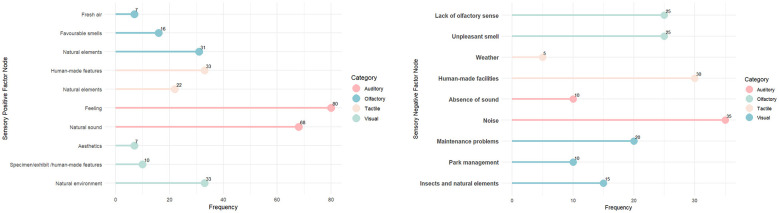
Sensory elements of positive and negative experiences for visually impaired groups.

To supplement the results of sensory mapping, NVivo software was used for textual analysis to identify the positive and negative sensory elements experienced by visually impaired visitors during their visits to the accessible sensory garden. Based on their textual descriptions of observations and feelings in specific areas of the garden, researchers conducted frequency counts and text coding in NVivo, as shown in Figure 8.

The analysis yielded several specific findings. First, visual perception showed the greatest differentiation between positive and negative experiences. Most of these visual elements were associated with natural features: visually impaired participants expressed strong positive feelings toward greenery, flowers, and lake views, while reporting negative experiences regarding issues of garden management and maintenance. Some participants with low vision paid greater attention to water features, green landscapes, as well as the yellow tactile paving and handrails within the garden, noting that these elements were helpful for their perceptual experience. In terms of vegetation, participants generally preferred brightly colored flowers, followed by lawns, while trees were least favored, primarily because their ornamental value was perceived as lower and they were less noticeable. Second, visually impaired participants tended to describe more positive than negative experiences with auditory, olfactory, and tactile stimuli. Many reported positive perceptions of natural sounds, animal calls, and background music. However, some expressed negative feedback on the bird calls broadcast in the Bird Science Corridor, noting that the pitch was too high. Third, in addition to the dominant role of auditory perception, participants also exhibited notable and vivid tactile experiences. Nevertheless, the investigation found that most participants did not actively touch the tactile installations within the garden. Last, regarding olfactory perception, participants reported relatively strong positive experiences with natural landscapes such as fresh air, plants, and trees, but negative reactions to odors such as paint and insect repellents.

## 4 Discussion

Taken together, the behavioral mapping results establish a descriptive baseline of who uses the accessible garden, where activities concentrate, and how users engage with different sensory spaces, thereby addressing the study's questions on user composition, spatial distribution, and use patterns. Building on this baseline, the SEEP interviews with visually impaired participants provide target-group evidence on how multisensory stimuli are perceived, which informs evidence-informed implications for optimizing sensory features and improving inclusive experiences in accessible gardens.

It is important to clarify the composition and scope of evidence regarding accessibility. The behavioral mapping dataset (*N* = 1,167) primarily captures general visitor patterns; consistent with Table 4, most observed users were classified as “unrestricted” (94.77%), and only a small proportion used mobility aids (e.g., wheelchairs, strollers, or white canes). Therefore, the quantitative mapping results should be interpreted as describing overall spatial–behavioral patterns rather than a direct evaluation of accessibility performance for disabled groups. Accessibility-related facilitators/barriers and multisensory experiences of visually impaired users are mainly supported by the SEEP interviews (*N* = 15) recruited via the Nanjing Association for the Blind. We explicitly avoid conflating general behavioral patterns with accessibility needs or evaluation criteria of visually impaired users. Future studies should expand on-site observations of disabled users and incorporate targeted accessibility metrics to strengthen representativeness and inference.

### 4.1 Multisensory experiences and behavioral patterns

Building on the behavioral mapping baseline and the SEEP interviews, this section interprets how different sensory-space types are associated with users' perceptual behaviors, and how visually impaired participants evaluate key sensory design elements.

Behavioral mapping indicates that the primary users of the accessible garden are adult women and older adults, whereas children tend to concentrate in areas characterized by visual and tactile features. This pattern aligns with prior evidence that landscape element preferences and activity profiles differ across age groups (Kabisch and Kraemer, 2020). Across sensory-space types, distinct behavioral profiles were observed. Visual spaces were dominated by passage and leisure/staying behaviors, reflecting their role as primary circulation routes combined with resting facilities. In contrast, auditory spaces were more frequently associated with social interaction behavior, suggesting that soundscape-related nodes can function as social attractors that facilitate interpersonal engagement (Akpinar, 2021; Jin et al., 2024).

Olfactory engagement occurred pre-dominantly as passive perception, typically triggered by the ambient diffusion of plants and other natural elements rather than deliberate seeking. Although users did not actively pursue olfactory stimuli, positive or negative experiences associated with these cues may be relevant to perceived restoration and affective responses reported in prior studies (Gorman, 2017; Hedblom et al., 2019; Yildirim et al., 2025; Spence, 2021). Tactile spaces, overall, were more closely associated with passage-related behaviors, consistent with the functional role of several tactile features (e.g., tactile paving) as wayfinding aids rather than destinations for prolonged stay (Tabassum, 2024). Texture variations along paths may enhance legibility and movement comfort but do not necessarily extend dwell time. Notably, tactile engagement at specific nodes with interactive installations was more likely to elicit active perceptual behaviors, supporting the value of actionable tactile affordances in promoting engagement (Qu and Ma, 2024).

The two datasets also enable an interpretive comparison of how different user groups may achieve social participation within the same sensory spaces. Behavioral mapping shows that auditory spaces are more frequently associated with social interaction behavior among general visitors, indicating that soundscape-related nodes can function as social attractors (Ríos-Rodríguez et al., 2021). SEEP interviews add a complementary perspective: visually impaired participants reported salient auditory cues (e.g., voice broadcasts) around the Bird Science Corridor and near the entrance to the core zone, suggesting that auditory features may support engagement through orientation, shared attention, and accompanied visits. At the same time, negative experiences were concentrated near the entrance to the extended area, where high background noise from the adjacent park gate interfered with sensory comfort. This juxtaposition highlights that the same auditory environment can facilitate interpersonal interaction for general visitors, while for visually impaired users its inclusiveness depends strongly on controllability, signal-to-noise conditions, and the balance between natural and artificial sounds. Importantly, this cross-group comparison is intended as contextual triangulation rather than a direct statistical comparison, given the different data sources and sample compositions.

### 4.2 Sensory environment optimization strategy

Based on the behavioral mapping results and SEEP interviews, the following implications are proposed to optimize multisensory environments in urban accessible gardens. These recommendations are grounded in (i) observed associations between sensory-space types and perceptual behaviors and (ii) visually impaired participants' reported positive and negative sensory experiences.

#### 4.2.1 Auditory environment: preserve social affordances while improving controllability and noise buffering

Behavioral mapping suggests that auditory spaces are more frequently associated with social interaction behavior, indicating their potential role as social attractors. Meanwhile, SEEP results show that negative experiences were concentrated near the entrance to the extended area, where high background noise interfered with sensory comfort. Therefore, auditory design should prioritize authentic, pre-dominantly natural soundscapes (e.g., water sounds and vegetation-mediated sounds) and reduce reliance on intrusive loudspeaker simulations. Voice broadcasts should be used sparingly, with improved control over volume, rhythm, and frequency to avoid masking other cues. Noise buffering should be strengthened at traffic-adjacent nodes and gateways (e.g., through denser, multi-layered planting and spatial setbacks) to improve the signal-to-noise conditions for both general visitors and visually impaired users.

#### 4.2.2 Tactile environment: move beyond wayfinding to actionable engagement at key nodes

In the behavioral mapping data, tactile spaces were overall associated with passage-related behaviors, consistent with the functional role of many tactile features (e.g., tactile paving and handrails) as wayfinding aids. However, node-based installations (e.g., play or interactive facilities) were more likely to elicit active perceptual behaviors. This suggests that tactile design should retain continuity and clarity for navigation, while adding safe and inviting tactile affordances at key nodes—such as touchable planting edges, multifunctional tactile devices, and interactive elements that encourage purposeful exploration—thereby supporting engagement rather than merely transit.

#### 4.2.3 Olfactory environment: strengthen positive natural cues and reduce avoidable negative odors

Olfactory perception in the garden was largely passive, and SEEP interviews indicated that visually impaired participants responded positively to natural odors (fresh air, plants, and trees) but reported negative experiences related to artificial odors (e.g., paint and insect repellents). Accordingly, aromatic planting can be used to form continuous and legible scent-based routes (e.g., along fragrance-related paths), while maintenance practices should minimize unpleasant odor sources near high-use routes and entrances. Where appropriate, subtle orientation cues (e.g., node markers) may complement olfactory continuity to support spatial legibility for visually impaired users.

#### 4.2.4 Visual environment: enhance low-vision legibility through contrast and structured planting

Because visual spaces dominate overall use patterns, improving visual legibility benefits a broad user base. Interview findings further suggest that low-vision users attend to high-contrast elements (e.g., tactile paving and handrails) that support recognition and wayfinding. Visual design should therefore strengthen edge definition and landmark readability at entrances, intersections, and resting nodes, using appropriate combinations of saturation and brightness. Planting design can enhance recognizability by emphasizing vertical layering (trees–shrubs–groundcovers) and seasonal contrast (e.g., foliage or bark colors that increase visibility), consistent with evidence that structured planting configurations are positively evaluated by visually impaired users (Zajadacz and Lubarska, 2020).

#### 4.2.5 System-level accessibility continuity and information delivery

SEEP narratives highlight the importance of gateways and transitions (e.g., core-zone entrance vs. extended-area entrance) for comfort and orientation. Accessible gardens should therefore ensure continuity of accessible design across entrances, path connections, and transitional zones, and provide multi-modal information delivery tailored to older adults and visually impaired users (Lin et al., 2025). A coordinated set of cues—controllable auditory information, continuous tactile guidance, and consistent olfactory landmarks—can improve spatial legibility and support inclusive, emotionally comfortable use.

### 4.3 Limitations and future research

Despite the contributions of this study, several limitations should be acknowledged. First, temporal coverage was constrained: behavioral mapping was conducted on two observation days in 2024 (May and October, 09:00–18:00). Although the number of recorded events was large (*N* = 1,167), the findings should be interpreted as seasonal snapshots rather than year-round estimates, as garden use may vary across weather conditions, holidays, peak visitation periods, and seasonal transitions. Data from the 2 days were aggregated to characterize overall spatial–behavioral patterns, and we did not conduct a formal season-stratified comparison. Given that seasonal conditions (e.g., temperature and phenology) may influence tactile engagement and olfactory perception, future studies should include repeated observations across multiple days and seasons to quantify seasonality effects.

Second, sampling and representativeness are limited in the qualitative component. The interview sample of visually impaired participants was relatively small (*N* = 15). Although recruitment through the Nanjing Association for the Blind facilitated access to participants with diverse visual status and onset types, the findings should be interpreted as exploratory and generalized with caution. Broader recruitment across multiple organizations and sites, combined with on-site observations of disabled users, would strengthen external validity.

Third because this study did not include physiological measurements or standardized restoration scales, any discussion of “psychological restoration” should be interpreted as behavioral and experiential correlates consistent with restorative potential, rather than as directly measured restorative mechanisms or causal pathways.

## 5 Conclusion

This study, grounded in behavior mapping, sensory compensation, and attention restoration theory, combined both quantitative and qualitative methods—including behavioral mapping and SEEP interviews—to systematically explore the relationship between perceptual spaces and user behaviors in urban accessible gardens, with particular attention to the sensory experience characteristics of visually impaired groups. While previous studies have applied similar approaches to examine sensory environments and behavioral patterns, their potential has not yet been fully realized. The results of this study demonstrate that the behavioral mapping method is highly applicable in revealing the associations between sensory stimuli and spatial usage patterns, effectively identifying users' behavioral preferences and interaction pathways within different sensory-dominant spaces. Furthermore, the findings highlight variations in sensory adaptation and preferences among visually impaired users in auditory, olfactory, and tactile spaces, underscoring their heightened sensitivity to environmental structures, facility maintenance, and sensory guidance mechanisms.

The study emphasizes that, when perceptual spaces in accessible gardens are properly designed and facilities are well maintained, these environments have the potential to play a more positive role in high-density urban settings, creating more equitable and open opportunities for outdoor activities across diverse ability groups. Beyond offering a behavioral map of general users' engagement with accessible garden spaces, this research also provides a spatiotemporal assessment of the multi-sensory experiences of visually impaired groups. The findings offer empirical evidence to inform the future design of sensory stimuli and interactivity in accessible gardens, while also contributing practical insights for fostering inclusivity in urban green space development. Strengthening human–environment perceptual interactions can enhance both the overall experiential quality and the functional value of urban outdoor spaces.

Given the inherent randomness of garden use, future research should further incorporate the perceptual evaluations of general users to comprehensively identify behavioral patterns and perceptual pathways across different groups within accessible gardens. In addition, future studies may introduce physiological indicators to quantify the restorative effects of sensory experiences and undertake cross-site comparative analyses to examine adaptive strategies for accessible gardens under diverse urban contexts, thereby advancing more diversified and inclusive approaches to urban green space construction.

## Data Availability

The raw data supporting the conclusions of this article will be made available by the authors, without undue reservation.
